# Plant-Mediated Enantioselective Transformation of Indan-1-one and Indan-1-ol. Part 2

**DOI:** 10.3390/molecules24234342

**Published:** 2019-11-27

**Authors:** Wanda Mączka, Katarzyna Wińska, Małgorzata Grabarczyk, Renata Galek

**Affiliations:** 1 Department of Chemistry, Wroclaw University of Environmental and Life Sciences, Norwida 25, 50-375 Wroclaw, Poland; 2 Department of Genetics, Plant Breeding and Seed Production, Wroclaw University of Environmental and Life Sciences Pl. Grunwaldzki 24A, 53-363 Wroclaw, Poland; renata.galek@upwr.edu.pl

**Keywords:** indan-1-one, indan-1-ol, biotransformation, reduction, oxidation, carrot, callus culture

## Abstract

The main purpose of this publication was to obtain the *S*-enantiomer of indan-1-ol with high enantiomeric excess and satisfactory yield. In our research, we used carrot callus cultures (*Daucus carota* L.), whereby the enzymatic system reduced indan-1-one and oxidized indan-1-ol. During the reaction of reduction, after five days, we received over 50% conversion, with the enantiomeric excess of the formed *S*-alcohol above 99%. In turn, during the oxidation of racemic indan-1-ol after 15 days, 36.7% of alcohol with an enantiomeric excess 57.4% *S*(+) remained in the reaction mixture. In addition, our research confirmed that the reactions of reduction and oxidation are competing reactions during the transformation of indan-1-ol and indan-1-one in carrot callus cultures.

## 1. Introduction

Indan-1-ol is example of secondary alcohol, which was observed as a semi-volatile product of *Lemna* sp. [1] and is also a component (8%) of floral essential oil of *Guettarda poasana* (*Rubiaceae*) [2]. The derivative of indanol, which was isolated from culture of *Ganoderma applanatum*, suppressed the growth of *Fusobacterium nucleatum* – a prominent member of the oral microflora implicated in periodontitis [3]. Analog of indanol: (1*S*, 2*R*)-1-amino-2-indanol is a key intermediate in the synthesis of Indinavir (Crixivan^®^), which acts as an HIV protease inhibitor in antiretroviral therapy. Although, it contains five chiral centers with 32 possible stereoisomers, only a single stereoconformation of Indinavir confers the desired therapeutic effect [4,5]. Chiral aminoindanol is a valuable substrate in preparation of other chiral auxiliaries used in asymmetric synthesis [4,6]. Indatraline, an analog of indanol, is used in the treatment of cocaine addiction [7]. In turn, PT285 and PT2877 are second-generation inhibitors of the hypoxia-inducible factor 2α (HIF-2α), key oncogenic driver in renal carcinoma [8].

The biocatalytic methods in obtaining single enantiomers of secondary alcohols are mainly based on two competitive reaction: reduction of ketones and oxidation of alcohols. Both reactions can be catalyzed by oxidoreductases, mainly alcohol dehydrogenases. These enzymes are NAD^+^- or NADP^+^-dependent. Therefore, cofactor recycling is an essential component of reaction mixture [9]. Desymmetrization of ketones, is highly significant in several processes because it allows 100% pure enantiomer in theoretical yield to be achieved. In turn, oxidative kinetic resolution is an efficient process involving the conversion of two enantiomers of alcohol in racemic mixtures into ketone at different rates, such that only one of the enantiomers remains. This process is limited in yield, because only one enantiomer undergoes a reaction, which results in theoretical maximum 50% of pure enantiomer [10].

Plant cell cultures have been employed for the enantioselective transformation of xenobiotics and have been shown to be good biochemical systems for enantioselective synthesis. Among the used cell and roots cultures, carrots seem to be very efficient for asymmetric reduction of prochiral ketones, not only in the form of crushed pulp [11,12,13,14,15,16,17,18,19,20,21,22] but also cell [17,23,24,25,26] and hairy roots cultures [27]. Based on literature date, [11,28,29,30,31] only few biocatalytic methods were used to obtain indan-1-ol enantiomers. The information presented above encouraged us to attempt research into the transformation of indan-1-one and indan-1-ol in carrot cell culture.

## 2. Results and Discussion

Both substrates, indan-1-one and indan-1-ol, used in biotransformation, are xenobiotic. Metabolism of foreign compounds in plants may undergo pathways that can be divided into two phases. During phase I metabolism, xenobiotics usually undergo biotransformation and the reaction of hydrolysis, hydroxylation, or other types of oxidation, producing intermediates with increased polarity or reactivity. Phase II metabolites are the conjugates of parent compound or phase I metabolites with polar biomolecules, such as amino acids, glutathione, or carbohydrates [32].

In turn, in our research we have proved that the carrot cell cultures obtained by us are able to carry out both reactions: oxidation of indan-1-ol and reduction of indan-1-one.

### 2.1. Reduction of Indan-1-one

Recently, we have developed an effective biotechnological method for obtaining *R*-(−)-indan-1-ol using ground Jerusalem artichoke pulp, where after 1 h over 50% conversion has been observed and the enantiomeric excess of unreacted *R*-alcohol was 100% [11]. During this transformation, highly stereo-selective oxidation of racemic alcohol was observed. In addition, looking for an effective way to obtain the second enantiomer, we noticed that the reduction of indan-1-one was highly stereo-selective, but with low yield. Our results are not consistent with the publication of Nagaki et al. [28], in which the authors showed that carrot cell cultures are a good biocatalyst only in the case of oxidation of indan-1-ol. During this biotransformation, they received 26% ketone after 25 days. Unfortunately, the authors did not provide results regarding the enantiomeric excesses obtained, and moreover, the authors pointed to the impossibility of carrying out indan-1-one reduction in carrot callus culture.

In the case of indan-1-one reduction in carrot culture in our research we have already received over 50% conversion after 5 days. The enantiomeric excess of alcohol, which were formed in the reaction, was above 99% ee. The formation of the *S*-enantiomer was preferred. (Table 1)

Our earlier publication regarding the reduction and oxidation reactions of these compounds, using the ground pulp of higher plants, indicated that this biocatalyst prefers the formation of the *S* enantiomer. However, in the case of crushed pulp it was possible to carry out the reaction only for 2 days. The low reduction efficiency observed during the transformation, using the ground pulp, resulted from the short reaction time.

By analyzing the results, we noticed that, during biotransformations in carrot callus cultures we did not observe much reaction progress between days 5 and 15. That is the reason why we decided to check the reaction over time. Samples were taken on days 1, 2, 3, 7, 8, 9, and 10 (Table 2). By analyzing the results, we noticed that the reactivity of the substrate has not changed rapidly after 7 days of the process.

During the research on the course of the biotransformation over time, we confirmed that only *S*-enantiomer is formed due to the highly stereo-selective reduction of the ketone carbonyl group. We observed such a high stereo-selectivity of reduction in our previous studies on the use of carrot enzymatic system in reduction of 3-methoxyacetophenone and 2-acetylnaphthalene [13,14]. Comparing the methodology presented by Nagaki et al. [28] we can assume that several factors may influence the results. First, the authors presented a different way of obtaining callus cultures. Among other things, they did not use growth regulators at the initial step and during biotransformation. The 2,4-D used by us as a growth regulator can affect the activity of oxygenases and dehydrogenases [33,34]. Both kind of enzymes could be responsible for this biotransformation.

Another factor that could affect the different course of these transformations was the use of a different medium in which the substrate was dissolved. Nagaki et al. [28] dissolved substrates in DMSO (dimethyl sulfoxide), which is characterized by low volatility. Therefore, this compound should be treated as a possible factor influencing the stereo-selectivity and course of biotransformation. The influence of this solvent on the course of enzymatic processes is also proven [35,36].

In our case, we used easily soluble acetone to dissolve the substrates. The solvent quickly left the reaction medium at 23 °C, and thus, did not significantly affect the course of the reaction.

### 2.2. Oxidation of Indan-1-ol

In the oxidation after just 5 days we observed a 43.7% of conversion with 27.1% of enantiomeric excess. (Table 3) The *S*-enantiomer was oxidized faster, what caused a slight excess of *R*-alcohol in reaction mixture. Therefore, it can be stated that the oxidation of the *S*-enantiomer was preferred at this stage of the process. However, after the next five days, the conversion increased by 15%, while the enantiomeric excess changed sharply. Mainly now the *S*-enantiomer of indan-1-ol remained in the mixture. After another 5 days, the conversion remained at a similar level. However, we received *S*-(+)-indan-1-ol with 57.4% of enantiomeric excess.

These results prompted us to more thoroughly check the reaction over time. As in the case of reduction, samples were taken after 1, 2, 3, 7, 8, 9, and 10 days of the reaction (Table 4). Between days 3 and 7 we observed a change in the configuration of the unreacted alcohol remaining in the reaction mixture - from *R* to *S*. Also, in this case, we observed a significant slow-down of reaction after 7 days. It should be mentioned that, during previous studies using ground vegetable pulp, we received 15.5% of conversion after 2 days and *S*-alcohol with 13.8% of enantiomeric excess remained in the mixture.

**Figure 1:**
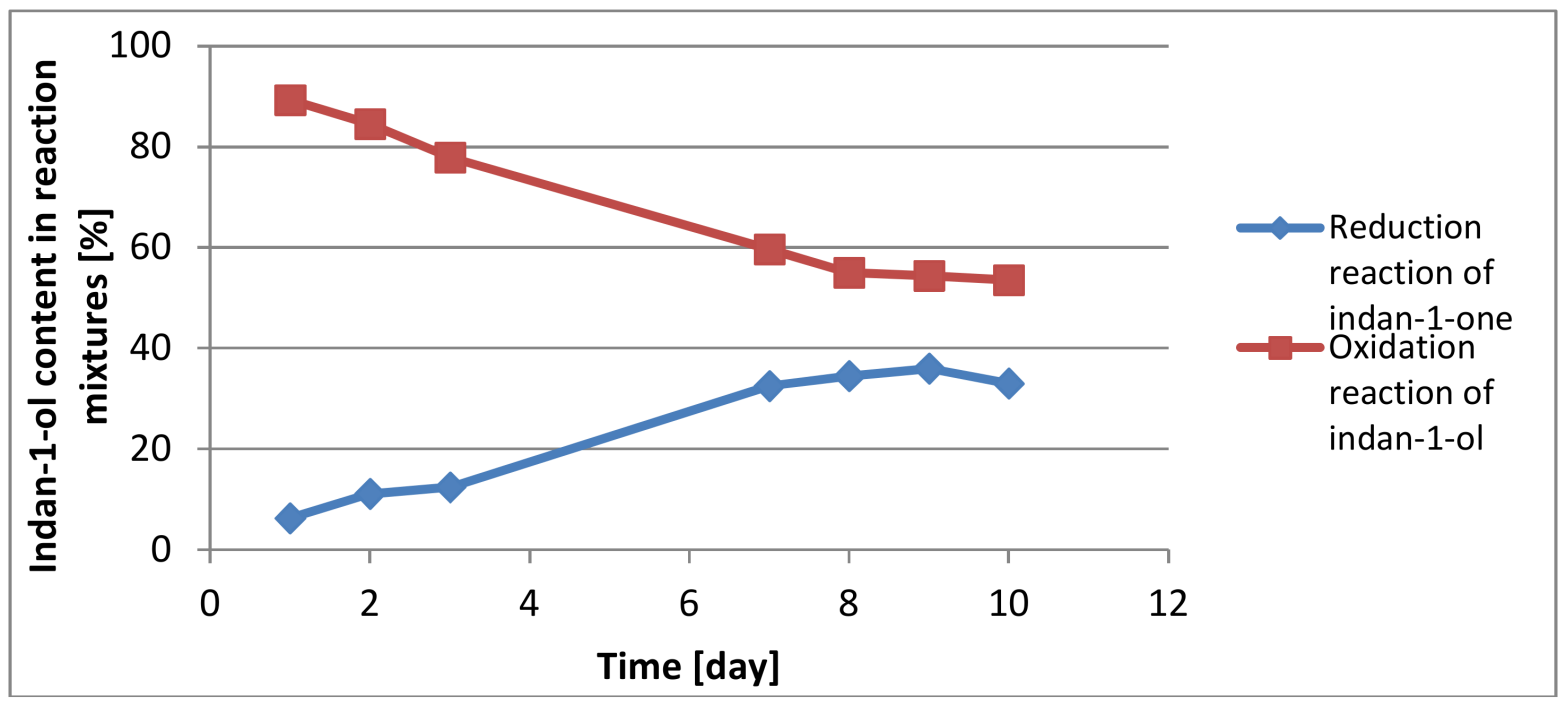
Comparison of indan-1-ol content over time during reaction of reduction and oxidation.

Therefore, we decided to compare the results that we received during the reduction of indan-1-one and oxidation of indan-1-ol using carrot callus cultures. The results are presented in Figure 1.

**Scheme 1:**
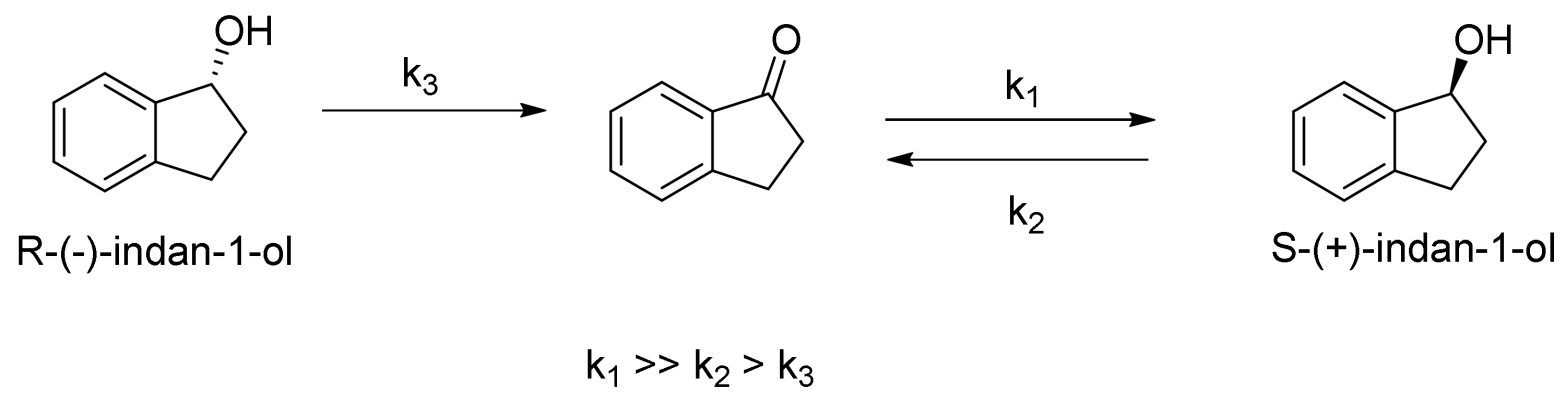
Comparison of reduction and oxidation.

After careful analysis of these reactions, we have found that oxidation and reduction reactions are part of the same equilibrium reaction. From indan-1-one, its reduction leads exclusively to the *S* configuration of the alcohol and ca. 40% conversion. For the same reason, the first step of the racemic alcohol oxidation involves the preferred conversion of the *S*-isomer of the alcohol, and the *R* enantiomer predominates. However, as the conversion proceeds, the ketone formed in this reaction activates its reduction to one pure *S*-enantiomer. After 10 days, the *S*-enatiomer predominates, because its formation is preferred during the reduction process. (Scheme 1).

## 3. Materials and Methods

### 3.1. Initiation and Stabilization of Callus Culture for Biotransformation

The fragments of *D. carota* roots were used for this experiment, which were bought from the local market. The healthy roots were washed with detergent water. They were washed with 70% ethanol for 1 min and surface sterilized by immersing them in the solution of HgCl_2_ (0.5%) for 10 min, and finally by rinsing three times (4, 10, 15) with sterile water. Fragments of roots were cultured on MS [37] medium supplemented with 0.5 NAA (1-naphthaleneacetic acid) mg/L at first two weeks. Callus proliferation was obtaining during cultured on MS medium solidified with 0.8% agar. Additionally, the medium contained 3% of sucrose and NAA at the rate of 0.5 mg/L and 1.5 mg/L in alternating culture every two to three weeks (total three mounts). pH of the medium was adjusted to 5.8 before autoclaving. Cultures were maintained at 23 °C ± 2 °C in the dark. The propagated callus was separated from the carrot root. Finally callus was cultivated into fresh liquid medium enriched with 2 mg/L of 2,4-D (2,4-dichlorophenoxyacetic acid) and the culture was continued for 1 month, passaging every 2 weeks until the desired amount of biomass was obtained. Suspension cultures were grown in 150 mL culture vessel containing 45 mL of medium on a rotary shaker at 100 rpm/min at 25 °C in the dark.

### 3.2. Method of Conducting Biotransformation

Biotransformations were carried out on MS with the addition of a 2,4-D as growth regulator in the amount of 2 mg/L. A totally of 10 mg of substrate dissolved in 200 μL acetone was added to the resulting suspension culture of carrot. After a specified time (5, 10 days, 15 days of culture), 5 mL of culture fluid was taken and 5 mL of chloroform was added. To break the cells, the sample was placed in an ultrasonic bath for 15 min. The chloroform layer was then collected and dried over anhydrous magnesium sulfate. The resulting extraction mixture was analyzed by gas chromatography. Each test was performed in duplicate. During study the course of biotransformation over time, samples were taken after 1, 2, 3, 7, 8, 9, and 10 days of biotransformation.

Separation of biotransformation products was done by column chromatography, the stationary phase was silicagel 60 with 70–230 mesh ASTM granulation and grain size 0.063–0.200 mm. As eluent for separation were used a mixture of hexane: chloroform in a ratio of 1:1.5.

### 3.3. Methods of Identification of the Biotransformation Products

The use of gas chromatography allowed to identify the composition of the post-reaction mixture. Analyzes were performed on a Varian CP-3380 apparatus (Varian, Agilent Technologies, Santa Clara, CA, USA). The carrier gas was hydrogen. The temperature program, which was used in GC analysis on the THERMO TR-5 (cross-linked 5% phenyl polisiloxane) capillary column (30 m × 0.32 mm × 1.0 µm), was as follows: injector 250 °C, detector (FID) 300 °C, column temperature: 100 °C (hold 2 min), 100–200 °C (rate 20 °C/min), 200–300 °C (rate 40 °C/min), 300 °C (hold 1 min). To determine the enantiomeric excess of indan-1-ol, GC analysis was performed using the chiral column Gamma DEX^TM^ 325 (30 m × 0.25 mm × 0.25 µm, Supelco) under the following conditions: injector 150 °C, detector (FID) 200 °C, column temperature: 110 °C (hold 35 min), 110–200 °C (rate 25 °C/min), 200 °C (hold 1 min). Some chromatograms of biotransformation are presented in supplementary data (Figures S1–S4).

## 4. Conclusions

Indan-1-ol enantiomers may find versatile use as chirons in the organic synthesis of other biologically active compounds. One of the biotechnological methods of obtaining them is the use of cell cultures for biotransformation. The indan-1-one reduction carried out by us in carrot callus culture was highly stereo-selective, allowing 53% of *S*-(+)-indan-1-ol to be obtained after five days of transformations. Further prolongation of the process resulted in a decrease in the reactivity of the substrate due to initiating a backwards reaction of the redox equilibrium. We also decided to study the oxidation of racemic indan-1-ol. After five days of transformation, 63% of ketone was obtained and the enantiomeric excess of unreacted indan-1-ol was 57% of *S*-(+)-alcohol. Investigating the course of this biotransformation, over time, clearly confirmed that, oxidation and reduction reactions compete with each other in the callus cultures of carrots, during the transformation of indanol and indanone.

## Figures and Tables

**Table 1:** Results of indan-1-one reduction using carrot callus culture.

Time [days]	Ketone [%]	Alcohol	
		[%]	ee [%]
5	46.7	53.3	99%S
10	56.3	43.7	99%S
15	55.6	44.4	99%S

Enantiomeric excess (ee) was determined by chiral GC (gas chromatography) analysis of the crude extract.

**Table 2:** Results of indan-1-one transformation using carrot callus culture over time

Time [days]	Ketone [%]	Alcohol [%]	
		[%]	ee [%]
1	93.7	6.3	99% S(+)
2	88.9	11.1	99% S(+)
3	87.6	12.4	99% S(+)
7	67.5	32.5	99% S(+)
8	65.5	34.5	99% S(+)
9	64	36.0	99% S(+)
10	66.8	33.2	99% S(+)

Enantiomeric excess (ee) was determined by chiral GC (gas chromatography)analysis of the crude extract.

**Table 3:** Oxidation of (±)-indan-1-ol using carrot callus culture

Time [days]	Ketone [%]	Alcohol	
		%	ee [%]
5	43.7	56.3	27.1 R(-)
10	61.1	38.9	34.4 S(+)
15	63.3	36.7	57.4 S(+)

Enantiomeric excess (ee) was determined by chiral GC (gas chromatography) analysis of the crude extract.

**Table 4:** Oxidation of (±) -indan-1-ol using carrot callus culture over time.

Time [days]	Ketone [%]	Alcohol	
		%	ee [%]
1	10.7	89.3	10.0 R(-)
2	15.5	84.5	13.8 R(-)
3	22.2	77.9	13.6 R(-)
7	50.3	49.7	6.5 S(+)
8	55.0	45.0	14.7 S(+)
9	55.6	44.4	21.9 S(+)
10	56.5	43.5	23.9 S (+)

Enantiomeric excess (ee) was determined by chiral GC (gas chromatography)analysis of the crude extract.

## Supplementary Materials

The following are available online at https://www.mdpi.com/1420-3049/24/23/4342/s1.

## References

[1] Catallo W.J., Shupe T.F., Eberhardt T.L. (2008). Hydrothermal processing of biomass from invasive aquatic plants. Biomass Bioenergy.

[2] Lawton R.O., Alexander L.D., Setzer W.N., Byler K.G. (1993). Floral essential oil of *Guettarda poasana* inhibits yeast growth. Biotropica.

[3] Fushimi K., Horikawa M., Suzuki K., Sekiya A., Kanno S., Shimura S., Kawagishi H. (2010). Applanatines A to E from the culture broth of *Ganoderma applanatum*. Tetrahedron.

[4] Gallou I., Senanayake C.H. (2006). *cis*-1-Amino-2-indanol in drug design and applications to asymmetric processes. Chem. Rev..

[5] Calitz C., Gouws C., Viljoen J., Steenekamp J., Wiesner L., Abay E., Hamman J. (2015). Herb-Drug pharmacokinetic interactions: Transport and metabolism of Indinavir in the presence of selected herbal products. Molecules.

[6] Lourenco N.M.T., Barreiros S., Afonso C.A.M. (2007). Enzymatic resolution of Indinavir precursor in ionic liquids with reuse of biocatalyst and media by product sublimation. Green Chem..

[7] Kameyama M., Siqueira F.A., Garcia-Mijares M., Silva L.F., Silva M.T.A. (2011). Indatraline: Synthesis and effect on the motor activity of Wistar rats. Molecules.

[8] Xu R., Wang K., Rizzi J.P., Huang H., Grina J.A., Schlachter S.T., Wang B., Wehn P.M., Yang H., Dixon D.D. (2019). 3-[(1*S*,2*S*,3*R*)-2,3-Difluoro-1-hydroxy-7-methylsulfonylindan-4-yl]oxy-5-fluorobenzonitrile (PT2977), a hypoxia-inducible factor 2α (HIF-2α) inhibitor for the treatment of clear cell Renal cell carcinoma. J. Med. Chem..

[9] Liu J., Wu S., Li Z. (2018). Recent advances in enzymatic oxidation of alcohols. Curr. Opin. Chem. Biol..

[10] Nasário F.D., Cazetta T., Moran P.J.S., Rodrigues J.A.R. (2016). Deracemization of 1-phenylethanol via tandem biocatalytic oxidation and reduction. Tetrahedron Asymmetry.

[11] Mączka W., Wińska K., Grabarczyk M., Galek R. (2019). Plant-Mediated enantioselective transformation of indan-1-one and indan-1-ol. Catalysts.

[12] Mączka W., Sołtysik D., Wińska K., Grabarczyk M., Szumny A. (2018). Plant-Mediated biotransformations of *S*(+)- and *R*(-)-carvones. Appl. Sci..

[13] Mączka W.K., Mironowicz A. (2002). Enantioselective hydrolysis of 1-aryl ethyl acetates and reduction of aryl methyl ketones using carrot, celeriac and horseradish enzyme systems. Tetrahedron Asymmetry.

[14] Mączka W.K., Mironowicz A. (2004). Enantioselective reduction of bromo- and methoxy-acetophenone derivatives using carrot and celeriac enzymatic system. Tetrahedron Asymmetry.

[15] Mączka W.K., Mironowicz A. (2004). Biotransformation of isoprenoids and shikimic acid derivatives by vegetable enzymatic system. Z. Naturforsch..

[16] Mączka W.K., Grabarczyk M., Wińska K., Anioł M. (2014). Plant-Mediated stereoselective biotransformation of phenylglyoxylic acid esters. Z. Naturforsch..

[17] Cordell G.A., Lemos T.L.G., Monte F.J.Q., de Mattos M.C. (2007). Vegetables as chemical reagents. J. Nat. Prod..

[18] Meshram S.H., Ramesh T., Nanubolu J.B., Srivastava A.K., Adari B.R., Sahu N. (2019). Green synthesis of enantiopure quinoxaline alcohols using Daucus carota. Chirality.

[19] Kazici H.C., Bayraktar E., Mehmetoglu Ü. (2017). Production of precursors for anti-Alzheimer drugs: Asymmetric bioreduction in a packed-bed bioreactor using immobilized *D. carota* cells. Prep. Biochem. Biotechnol..

[20] Omori A.T., Lobo F.G., Gonçalves do Amaral A.C., de Oliveira C.S. (2016). Purple carrots: Better biocatalysts for the enantioselective reduction of acetophenones than common orange carrots (*D. carota*). J. Mol. Catal. B Enzym..

[21] Yadav J.S., Nanda S., Thirupathi Reddy P., Bhaskar Rao A. (2002). Efficient enantioselective reduction of ketones with *Daucus carota* root. J. Org. Chem..

[22] Utsukihara T., Horiuchi A. (2019). Production of chiral aromatic alcohol by acetophenone and 1-arylethanol derivatives using vegetables. J. Chem..

[23] Mironowicz A., Kromer K. (1998). Apple-Tree shoots and transformed carrot and apple roots used as biocatalysts in enantioselective acetate hydrolysis, alcohol oxidation and ketone reduction. Collect. Czech. Chem. Commun..

[24] Naoshima Y., Akakabe Y. (1991). Biotransformation of aromatic ketones with cell cultures of carrot, tobacco and Gardenia. Phytochemistry.

[25] Akakabe Y., Naoshima Y. (1994). Biotransformation of acetophenone with immobilized cells of carrot, tobacco and Gardenia. Phytochemistry.

[26] Baskar B., Ganesh S., Lokeswari T.S., Chadha A. (2004). Highly stereoselective reduction of 4-aryl-2-oxobut-3-enoic carboxylic esters by plant cell culture of *Daucus carota*. J. Mol. Catal. B: Enzym..

[27] Caron D., Coughlan A.P., Simard M., Bernier J., Piché Y., Chênevert R. (2005). Stereoselective reduction of ketones by *Daucus carota* hairy root cultures. Biotech. Lett..

[28] Nagaki M., Soma N., Ono K., Yamanouchi K., Tsujiguchi T., Kawakami J., Chounan Y. (2019). Biotransformation of indanol, fluorenol and their analogs using tissue-cultured cells and their antimicrobial activity. Trans. Mat. Res. Soc..

[29] Bennamane M., Razi S., Zeror S., Aribi-Zouioueche L. (2018). Preparation of chiral phenylethanols using various vegetables grown in Algeria. Biocatal. Agric. Biotechnol..

[30] Uzura A., Katsuragi T., Tani Y. (2001). Conversion of various aromatic compounds by resting cells of Fusarium moniliforme strain MS31. J. Biosci. Bioeng..

[31] Stampfer W., Kosjek B., Faber K., Kroutil W. (2003). Biocatalytic asymmetric hydrogen transfer employing Rhodococcus ruber DSM 44541. J. Org. Chem..

[32] Wu X., Fu Q., Gan J. (2016). Metabolism of pharmaceutical and personal care products by carrot cell cultures. Environ. Pollut..

[33] Song Y. (2014). Insight into the mode of action of 2,4-dichlorophenoxyacetic acid (2,4-D) as an herbicide. J. Integr. Plant Biol..

[34] Pazmino D.M., Rodriguez-Serrano M., Romero-Puertas M.C., Archilla-Ruiz A., Del Rio L.A., Sandalio L.M. (2011). Differential response of young and adult leaves to herbicide 2,4-dichlorophenoxyacetic acid in pea plants: Role of reactive oxygen species. Plant Cell Environ..

[35] Chan D.S.-H., Matak-Vinković D., Coyne A.G., Abell C. (2016). Insight into protein conformation and subcharging by DMSO from native ion mobility mass spectrometry. Chem. Select..

[36] Könst P., Merkens H., Kara S., Kochius S., Vogel A., Zuhse R., Holtmann D., Arends I.W.C.E., Hollmann F. (2012). Oxidation von Aldehyden mit Alkoholdehydrogenasen. Angew. Chem. Int. Ed..

[37] Murashige T., Skoog F. (1962). A revised medium for rapid growth and bioassays with tobacco tissue cultures. Physol. Plant..

