# Supplementary material for: Plant-Mediated Enantioselective Transformation of Indan-1-one and Indan-1-ol. Part 2
Source: Molecules. 2019 Nov 27;24(23):4342. doi: 10.3390/molecules24234342 (PMC6930634; doi:10.3390/molecules24234342)
Supplement: Supplementary file 1 [file molecules-24-04342-s001.pdf]

Supplementary materials

Plant-mediated enantioselective transformation of indan-1-one and indan-1-ol. Part 2.

Wanda Mączka, Katarzyna Wińska, Małgorzata Grabarczyk and Renata Galek

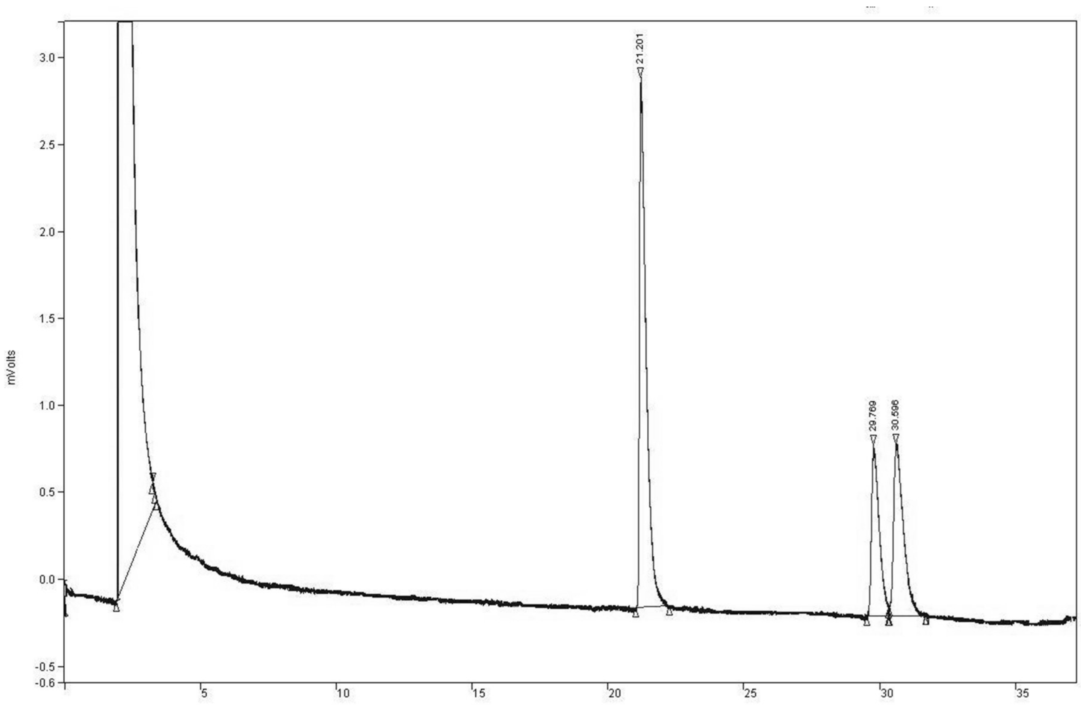

Figure S1. Chromatogram of standards: 21.120 min. – indan-1-one, 29.769 min. – S-(+)-indan-1-ol, 30.596 min. – R-(–)-indan-1-ol.

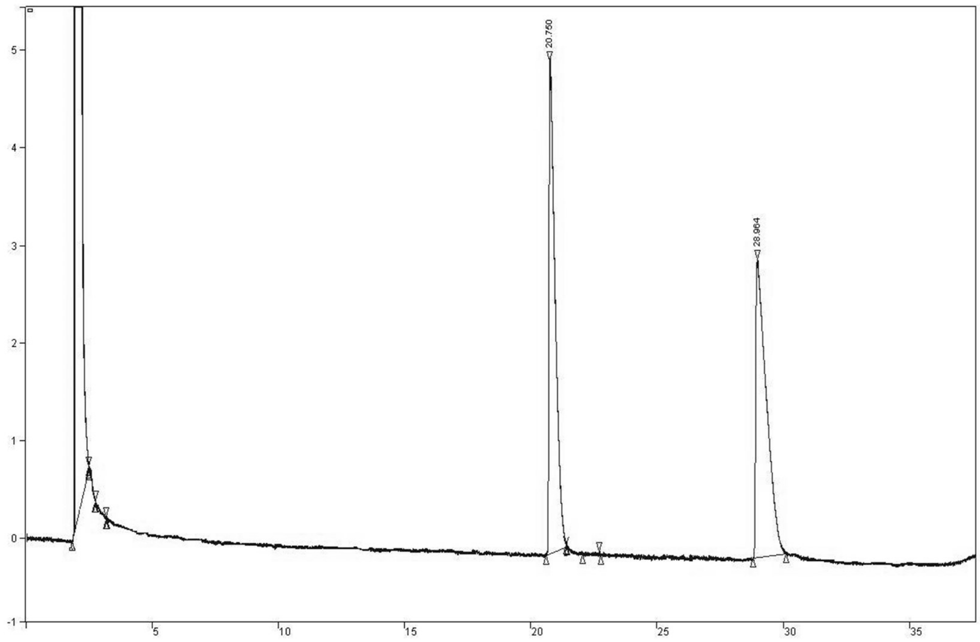

Figure S2. Chromatogram of biotransformation of indan-1-one by carrot cell culture.

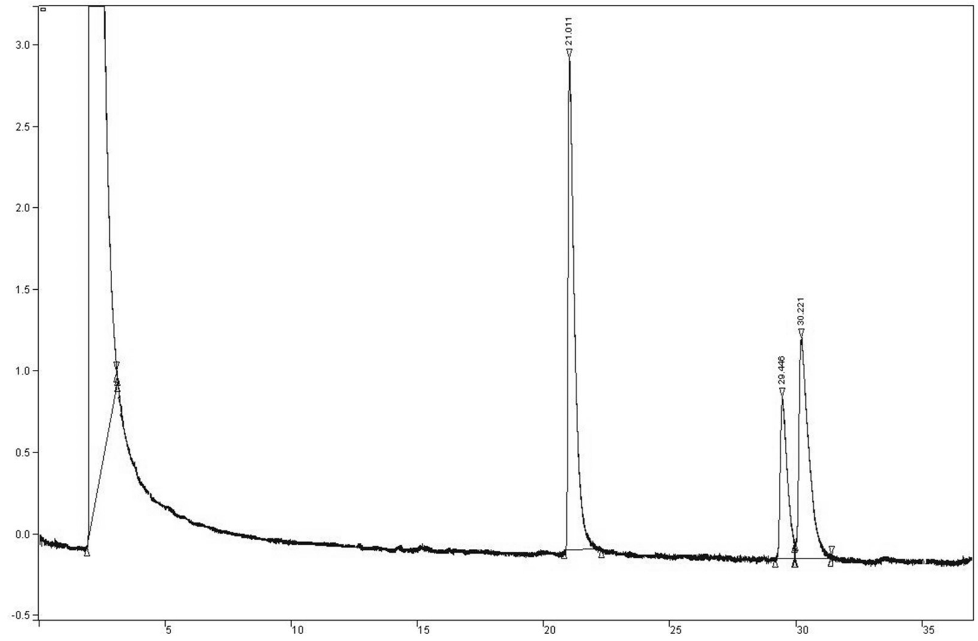

Figure S3. Chromatogram of oxidation of indan-1-ol.

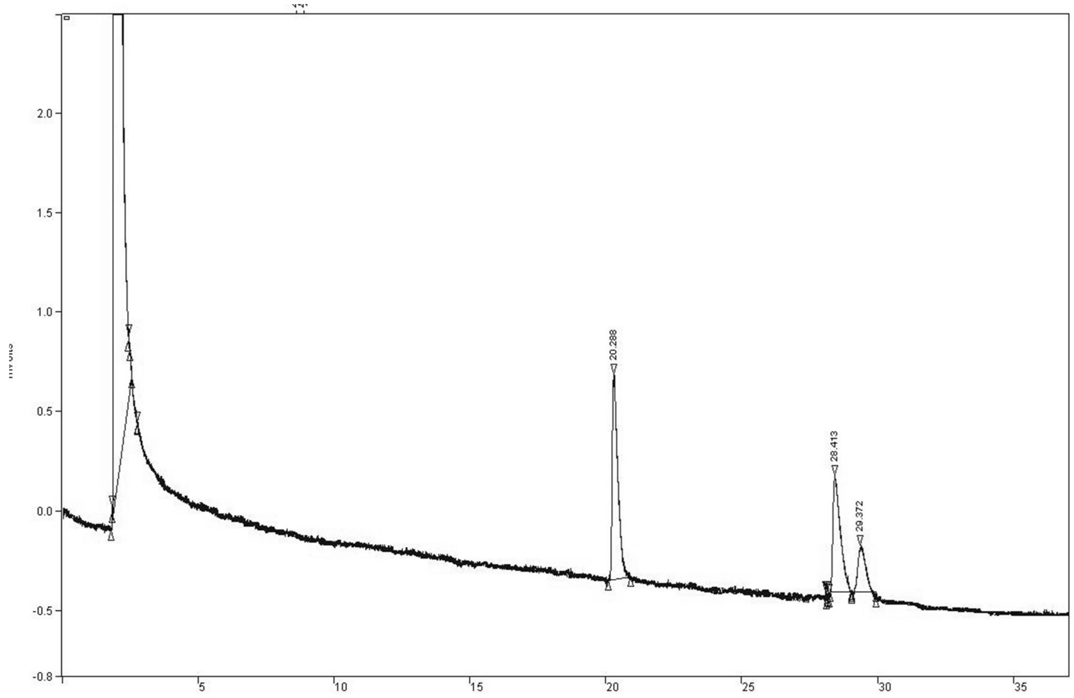

Figure S4. Chromatogram of oxidation of indan-1-ol.
